# Pharmacogenetics of Statin-Induced Myopathy: A Focused Review of the Clinical Translation of Pharmacokinetic Genetic Variants

**DOI:** 10.4172/2153-0645.1000128

**Published:** 2014-04-23

**Authors:** Jasmine A Talameh, Joseph P Kitzmiller

**Affiliations:** Center for Pharmacogenomics, The Ohio State University, Columbus, OH, USA

**Keywords:** Statin, Pharmacogenetics, Statin-Induced Myopathy, Pharmacokinetics

## Abstract

Statins are the most commonly prescribed drugs in the United States and are extremely effective in reducing major cardiovascular events in the millions of Americans with hyperlipidemia. However, many patients (up to 25%) cannot tolerate or discontinue statin therapy due to statin-induced myopathy (SIM). Patients will continue to experience SIM at unacceptably high rates or experience unnecessary cardiovascular events (as a result of discontinuing or decreasing their statin therapy) until strategies for predicting or mitigating SIM are identified. A promising strategy for predicting or mitigating SIM is pharmacogenetic testing, particularly of pharmacokinetic genetic variants as SIM is related to statin exposure. Data is emerging on the association between pharmacokinetic genetic variants and SIM. A current, critical evaluation of the literature on pharmacokinetic genetic variants and SIM for potential translation to clinical practice is lacking. This review focuses specifically on pharmacokinetic genetic variants and their association with SIM clinical outcomes. We also discuss future directions, specific to the research on pharmacokinetic genetic variants, which could speed the translation into clinical practice. For simvastatin, we did not find sufficient evidence to support the clinical translation of pharmacokinetic genetic variants other than *SLCO1B1*. However, *SLCO1B1* may also be clinically relevant for pravastatin- and pitavastatin-induced myopathy, but additional studies assessing SIM clinical outcome are needed. CYP2D6*4 may be clinically relevant for atorvastatin-induced myopathy, but mechanistic studies are needed. Future research efforts need to incorporate statin-specific analyses, multi-variant analyses, and a standard definition of SIM. As the use of statins is extremely common and SIM continues to occur in a significant number of patients, future research investments in pharmacokinetic genetic variants have the potential to make a profound impact on public health.

## Background

Cardiovascular disease is the leading cause of morbidity and mortality in the United States. An estimated 83.6 million American adults (>1 in 3) have cardiovascular disease [1]. Statins, or 3-hydroxy-3-methylglutaryl-coenzyme A (HMG CoA) reductase inhibitors, are highly effective in reducing the risk for major cardiovascular events by lowering low-density lipoprotein cholesterol (LDL-C). Specifically, statins reduce the risk of major cardiovascular events by approximately 20% per mmol/L (38 mg/dL) reduction in LDL-C [2]. Statins are the most commonly prescribed class of drugs in the United States; greater than 25% of Americans over the age of 45 use a statin [3]. Moreover, the number of Americans treated with statins is expected to increase as a result of the new cholesterol treatment guidelines [4]. However, many patients treated with statins experience adverse effects, often leading to dose-lowering, non-compliance, and even discontinuation of therapy. The most common adverse effect of the statins is statin-induced myopathy (SIM). Clinical symptoms of SIM can include muscle pain, soreness, and/or weakness and are often accompanied by increases in creatine kinase (CK) levels. The true frequency of SIM has been widely debated. Clinical trial data suggest the frequency of SIM to be lower than 5% [5], but frequencies of 60% and 25% have been reported in an observational study of former and current statin users, respectively [6]. As clinical trials are implemented in select patient populations and typically utilize a run-in period that likely excludes participants who are intolerant to statins, the SIM frequency suggested by clinical trial data may be largely underestimated. Rhabdomyolysis is the rarest (≤ 0.1% frequency) form of SIM, but it is the most severe and sometimes fatal. Patients will continue to experience SIM at unacceptably high rates or will continue to experience unnecessary cardiovascular events (as a result of discontinuing or decreasing their statin therapy) [7–9] until strategies for predicting or mitigating SIM are identified.

Numerous clinical factors have been associated with SIM including age, gender, body-mass-index, exercise, comorbidities, duration of statin use, statin dose, type of statin, and the use of concomitant medications [6,10,11]. One of the most important risk factors for SIM, which could be the result of the aforementioned clinical factors, is increased exposure (systemic or intra-organ concentrations) to the statin and its metabolites [12]. Variants in the genes involved in statin pharmacokinetics (*i.e.,* statin metabolizing enzymes and transporters) affect statin exposure *in vivo* and have been further linked to SIM clinical outcome. Therefore, pharmacogenetic testing of pharmacokinetic genetic variants is one possible strategy for predicting or mitigating SIM. Indeed, the data supporting the association between a variant (rs4149056; T521C; Val174Ala) in *SLCO1B1* (the gene encoding the solute carrier organic anion transporter family member 1B1) and simvastatin-induced myopathy was so strong that the Clinical Pharmacogenetics Implementation Consortium (CPIC) wrote guidelines for simvastatin therapy based on *SLCO1B1* T521C genotype [13]. However, a current, critical evaluation of the literature on other pharmacokinetic genetic variants for translation to clinical practice is lacking. Other recent articles have reviewed genetic variants associated with SIM [14–16], but they did not focus specifically on pharmacokinetic genetic variants, the caveats specific to research on pharmacokinetic genetic variants, or the potential for pharmacokinetic genetic variants to be translated into clinical practice. Our review intends to address those specific foci.

## Methods

**Figure 1:**
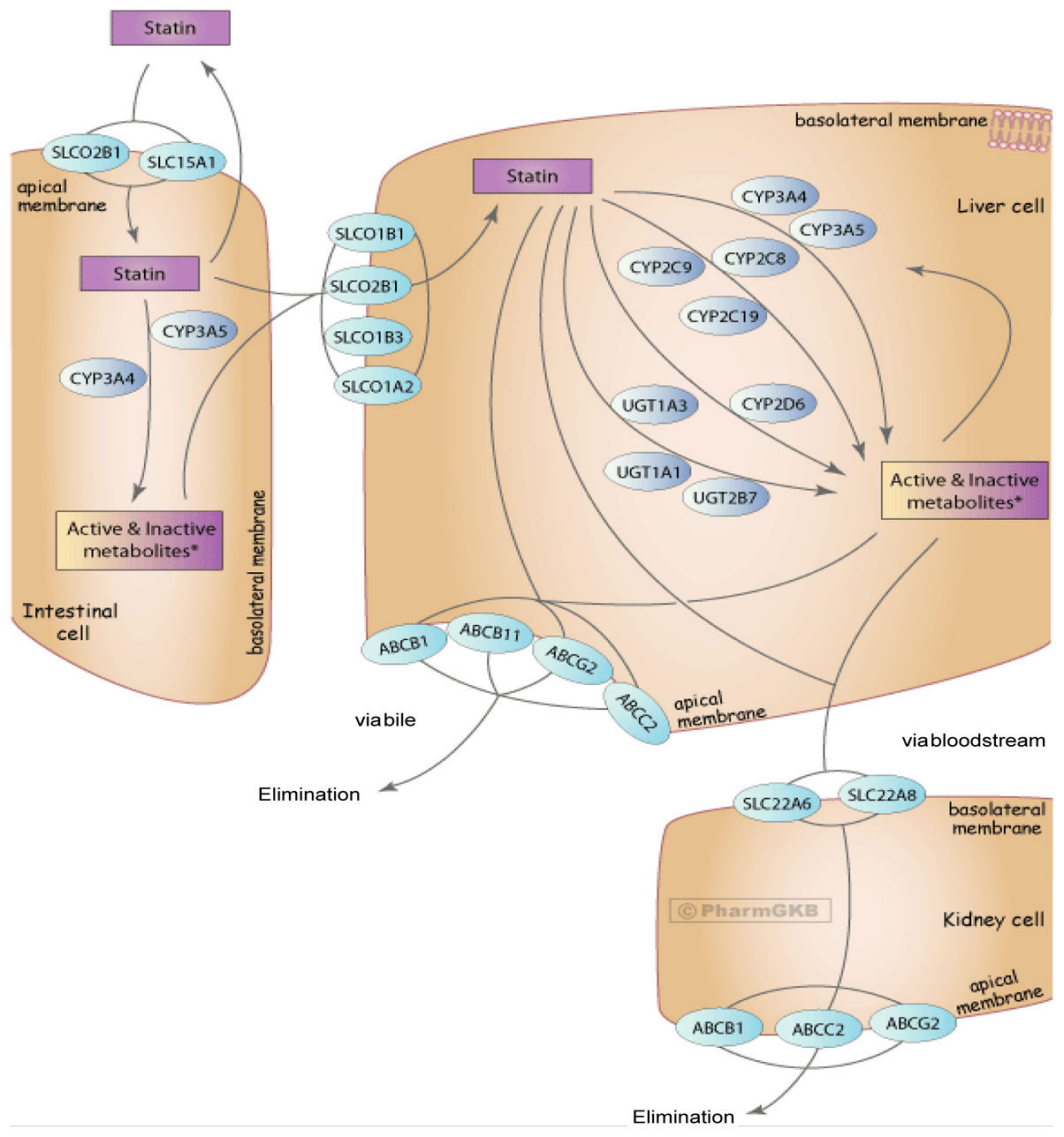
Representation of the superset of all genes involved in the transport, metabolism and clearance of statin class drugs. ^©^PharmGKB. (Reproduced with permission from the Pharmacogenomics Knowledge Base [PharmGKB] and Stanford University.)

Pharmacogenetic studies of SIM clinical outcome and pharmacokinetic genetic variants were identified in the PubMed database through March 11, 2014 by combining the following search terms: statin, gene, genetic, myopathy, and myalgia. These search terms were also used in the Cochrane Library and Centre for Reviews and Dissemination electronic databases, but no additional articles on this topic were identified in those databases. Studies were also identified from the reference lists of articles. Studies were included in this review if they analyzed genes involved in the pharmacokinetics of statins (Figure 1 [17]; Table 1) and SIM clinical outcome. Pharmacokinetic genetic variants were the focus of this review because SIM is related to statin exposure [12,18], and one pharmacokinetic genetic variant (*SLCO1B1* T521C) has CPIC guidelines for clinical translation [13]. For a more general review of other potential mechanisms of SIM (*e.g*., muscle pathology), the reader is referred elsewhere [14]. Because our focus is on the clinical translation of pharmacokinetic genetic variants, SIM clinical outcomes (as opposed to pharmacokinetic endpoints) were chosen for this review. Pharmacokinetic genetic variants associated with SIM clinical outcome are more likely to be translated into clinical practice than pharmacokinetic endpoints. SIM clinical outcome has various definitions [19], but studies were included if they include muscle symptoms and/or elevations in plasma levels of creatine kinase (CK). Studies with pharmacokinetic endpoints (*e.g.,* the association between pharmacokinetic genetic variants and statin concentrations) were only searched and reviewed in the context and absence of SIM clinical outcome data. Other types of studies (with limited potential for clinical translation) that were excluded were case reports, animal studies, *in vitro* studies, and those focused on cerivastatin as it is no longer on the market in the U.S. Only studies published in English were reviewed. Research studies published only in abstract form were not reviewed because the methodological details could not be evaluated. As this is a focused review on SIM, we did not review data on other statin-induced toxicities (*e.g.* abnormal liver function tests) or statin efficacy (*e.g.* LDL-C lowering). We have reviewed the data by statin because each statin has a unique pharmacokinetic profile (*i.e.,* each statin has different substrate specificities for metabolizing enzymes and transporters).

### Simvastatin

Eighteen studies met the inclusion and exclusion criteria described above (Table 2), and 13 of those studies included patients receiving simvastatin. Because clinical guidelines have already been written for *SLCO1B1* T521C [13], our goal was to evaluate the data for other pharmacokinetic genetic variants that may have potential for clinical translation related to simvastatin-induced myopathy.

In two studies, *CYP2D6* genotype was marginally associated with simvastatin-induced myopathy, and in two more studies there was no association. Mulder et al. [20] performed a prospective trial of 88 patients with primary or secondary hypercholesterolemia that were started on 10 mg of simvastatin and titrated up to a dose of 40 mg over several weeks. Although not statistically significant, the proportion of patients that discontinued simvastatin due to intolerability increased as their number of mutated *CYP2D6* alleles increased. Frudakis et al. [21] found that the frequency of the CYP2D6*4 allele was slightly higher in simvastatin-treated cases of SIM (49%; n=61) than in controls (36%; n=108; p=0.067). However, a study by Zuccaro et al. [22] that included 24 simvastatin-treated patients and a study by Voora et al. [23] that included 162 simvastatin-treated patients did not find a significant association between *CYP2D6* genotype and SIM. Notably, Zuccaro et al. [22] did not perform a simvastatin-specific analysis, and the power to detect an association may have been diluted because statins not metabolized by *CYP2D6* (*e.g.* pravastatin) were included. We did not find any studies that demonstrated an association between *CYP2D6* genotype and simvastatin exposure, but *in vitro* data suggests that simvastatin acid (the active metabolite of the parent form, simvastatin lactone) has a similar affinity for *CYP2D6* as *CYP3A4* [24]. However *CYP2D6* is not involved in the metabolism of the parent drug, simvastatin lactone [25]. It is unknown whether the parent drug, simvastatin lactone, and/or its major metabolite, simvastatin acid, cause simvastatin-induced myopathy. Data suggests that atorvastatin lactone, not atorvastatin acid, contributes to atorvastatin-induced myopathy [12]. Like atorvastatin, simvastatin is present *in vivo* in both acid and lactone forms. If the lactone form of simvastatin is the causative agent for simvastatin-induced myopathy, *CYP2D6* genotype is not likely to associate with SIM. This hypothesis is supported by the lack of association between *CYP2D6* and SIM reported by Zuccaro et al. [22] and Voora et al. [23]. Because the findings by Mulder et al. [20] and Frudakis et al. [21] were not statistically significant, or supported by the studies reported by Zuccaro et al. [22] or Voora et al. [23] or by pharmacokinetic data, *CYP2D6* variation is unlikely to be translated for clinical use of simvastatin.

Two studies found a significant association, albeit with discordant results, between *ABCB1* variants and SIM clinical outcome in simvastatin-treated patients. Fiegenbaum et al. [26] performed a prospective trial of 146 patients treated with 20 mg simvastatin for 6 months. They found a reduced frequency of the *ABCB1* T-non-G-T haplotype (C1236T-G2677T/A-C3435T) in patients having experienced myalgia compared to patients who did not experience myalgia (20% vs. 41%; p=0.03). However, a case-control study reported by Ferrari et al. [27] of 66 patients (23 treated with simvastatin) found increased frequencies of the *ABCB1* 1236T and 3435T alleles in the patients that experienced CK elevations. Both Keskitalo et al. [28] and Zhou et al. [29] reported no association of *ABCB1* C1236T-G2677T/A-C3435T haplotype with the pharmacokinetics of simvastatin lactone, but Keskitalo et al. [28] reported increased simvastatin acid exposure with the TTT/TTT diplotype. Moreover, *in vitro* data on the function of *ABCB1* C1236T-G2677T/A-C3435T haplotype is inconclusive. Because the SIM clinical outcome and *ABCB1* data by Fiegenbaum et al. [26] and Ferrari et al. [27] are discordant, and the pharmacokinetic and *in vitro* data are inconclusive, the clinical translation of *ABCB1* genotyping for simvastatin-induced myopathy currently seems unlikely.

The SEARCH Collaborative Group and Voora et al. [23] evaluated other pharmacokinetic genetic variants when evaluating *SLCO1B1* genotypes for simvastatin-induced myopathy [18]. The SEARCH Collaborative Group performed a genome-wide association study (GWAS) (n=175 in discovery cohort and n=16,664 in the replication cohort), and Voora et al. [23] performed a candidate gene association study (n=162 treated with simvastatin) of *SLCO1B1* and common variants in cytochrome P450 enzymes. In both of these studies, only *SLCO1B1* genotype was statistically significant. The power of the study by the SEARCH Collaborative Group was limited because of the correction for multiple comparisons in the discovery cohort. Therefore, other potentially important pharmacokinetic genes may have gone undetected. The SEARCH Collaborative Group states that the existence of other genetic variants that carry a relative risk of myopathy of 2 to 4 cannot be ruled out by their analysis. Voora et al., [23] however, did not find associations with SIM for pharmacokinetic genes other than *SLCO1B1* even though their analysis had greater power (relative risk of 2 or greater) secondary to the candidate gene design. This suggests *SLCO1B1* may be the only pharmacokinetic gene significantly important in simvastatin-induced myopathy. Alternatively, *SLCO1B1* may be the only pharmacokinetic gene with a clinically significant effect size. The *CYP2D6* associations reviewed above were not statistically significant, and the *ABCB1* SIM clinical outcome associations were inconclusive. Moreover, a GWAS by Isackson et al. [30] of 229 patients with severe SIM, in which 20% were treated with simvastatin, did not find a significant association with any pharmacokinetic genetic variants. Therefore, the likelihood of translation into clinical practice for pharmacokinetic genes other than *SLCO1B1* for simvastatin is minimal.

### Atorvastatin

Fourteen of the 18 studies investigating pharmacokinetic genetic variants and SIM clinical outcome (Table 2) included patients receiving atorvastatin. Although more studies included patients receiving atorvastatin than simvastatin, the currently available data for atorvastatin has not been strong enough to prompt the writing of any clinical guidelines by CPIC or to prompt the FDA to require the inclusion of pharmacokinetic genetic information into drug labeling of atorvastatin. Notably, other genetic information, regarding the genetic disorder familial hypercholesterolemia, has been incorporated into the FDA labeling for atorvastatin [31].

The evidence supporting *SLCO1B1* T521C in simvastatin-induced myopathy was very strong, and because atorvastatin is also a substrate for *SLCO1B1,* this gene has been widely studied for an association with atorvastatin-induced myopathy. The data supporting an association of *SLCO1B1* variation with atorvastatin-induced myopathy, however, is not nearly as strong as it is for simvastatin. Ten studies have tested the association between *SLCO1B1* variants and atorvastatin-induced SIM clinical outcome. Three found a significant association, but only one study performed an atorvastatin-specific analysis. Donnelly et al. [32] and Ferrari et al. [27] reported a significant association between *SLCO1B1* variants and SIM clinical outcome, but they did not perform an atorvastatin-specific analysis. Their studies included simvastatin and atorvastatin. Therefore, it cannot be determined whether the associations were driven solely by the simvastatin-treated patients. Puccetti et al. [33] reported a significant association between *SLCO1B1* variants and atorvastatin intolerance (OR=2.7; 95% CI=1.3–4.9; p < 0.001), but this data is preliminary and published only as an editorial letter. Acknowledging the strength of this association, this data is preliminary and the association may not remain statistically significant when the final study analysis is completed. Hermann et al. [12], Carr et al. [34], Voora et al. [23] and Brunham et al. [35] performed atorvastatin-specific analyses but did not find a significant association between *SLCO1B1* variation and atorvastatin-induced myopathy. A meta-analysis performed by Carr et al. [34], also reported no association. *SLCO1B1* variation does affect atorvastatin pharmacokinetics [36], but the effect size for atorvastatin is not as large as it is for simvastatin. The difference in exposure in atorvastatin-treated subjects homozygous for *SLCO1B1* T521C was 144%, and the difference for simvastatin 221% [37]. This difference in the pharmacokinetic effects of *SLCO1B1* on atorvastatin and simvastatin may explain the differences in SIM clinical outcome between *SLCO1B1* variation and those statins. Currently, the clinical outcome data as a whole does not support the influence of *SLCO1B1* variation on atorvastatin-induced myopathy for clinical translation.

Other pharmacokinetic genes that have been associated with atorvastatin-induced myopathy include *CYP3A5*, *CYP2D6*, and *ABCB1*. In a case-only analysis, Wilke et al. [38] found a significant association between CYP3A5*3 and the degree of CK elevation in 68 patients with atorvastatin-induced myopathy that were not treated with gemfibrozil or niacin. The clinical significance of this finding is limited, as the difference in CK between CYP3A5*3 homozygous and heterozygous patients was only 25% when including patients treated with gemfibrozil or niacin. The difference was larger when analyzing only the patients not treated with gemfibrozil or niacin (71%), but excluding those patients limits the generalizability of the results. Moreover, CK levels do not correlate well with myopathy symptoms. In their case-control analysis, Wilke et al. [13] did not find a significant difference in the frequency of CYP3A5*3 between the cases and controls, which is consistent with three other studies [12,21,22]. These other studies did not perform a case-only analysis like Wilke et al. [13], but the preponderance of negative case-control findings makes it seem unlikely that CYP3A5*3 is clinically meaningful for atorvastatin. These negative results for atorvastatin and SIM clinical outcome are supported by the pharmacokinetic data from Shin et al. [39]. They found a statistically significant association between CYP3A5*3 and atorvastatin exposure, but the difference between genotypes was small (36%). DeGorter et al. [36] demonstrate that atorvastatin plasma concentrations were significantly associated with CYP3A activity, as assessed by a CYP3A activity marker but not by genotype. Therefore, factors affecting CYP3A activity other than genotype (*e.g.,* concomitant use of CYP3A inhibitors) may prove to be clinically important for predicting atorvastatin-induced myopathy.

Frudakis et al. [21] reported a significant association between atorvastatin-induced muscle effects and CYP2D6*4 in discovery (n=106) and blind validation (n=157) cohorts. This finding was unexpected as atorvastatin is not known to be metabolized by *CYP2D6*. Interestingly, *CYP2D6* poor metabolizer/intermediate metabolizer classification was not associated. The mechanism of CYP2D6*4, therefore, may not be related to atorvastatin metabolism. Zuccaro et al. [22] and Voora et al. [23] studied CYP2D6*4 and atorvastatin-induced adverse effects but did not find a significant association. Although we could not find any data to support an association of CYP2D6*4 with atorvastatin pharmacokinetics, *in vitro* data suggests that atorvastatin can inhibit *CYP2D6* activity [40]. Because of the inconsistent results in the clinical outcome studies and the lack of pharmacokinetic data, the mechanism by which CYP2D6*4 affects atorvastatin-induced myopathy must be investigated before clinical translation should be pursued.

Hoenig et al. [41] reported a significant association between the *ABCB1* C3435T variant and atorvastatin-induced myalgia in 98 patients, but the clinical significance of this finding is limited because of the small number of events, the limited clinical discrimination, and the lack of replication. The *ABCB1* 3435T allele was more frequent in patients with atorvastatin-induced myalgia (80% vs 62%; p=0.043), but only 10 genotyped patients reported myalgia. Clinical discrimination was limited because 86% of 3435TT patients did not have myalgia. The findings by Ferrari et al. [27] are consistent with those by Hoenig et al. [41] in that the frequency of the *ABCB1* 3435T allele increased in the patients with CK elevations (p=0.013). Hermann et al. [12] also studied *ABCB1* C3435T but did not find a difference in allele frequencies between atorvastatin-treated cases and controls. DeGorter et al. [36] did not find an association between *ABCB1* G2677T/A (which is in linkage disequilibrium with C3435T) and atorvastatin levels in a cohort study representative of real-world clinical practice, but pharmacokinetic data supports the associations found by Hoenig et al. [42] and Ferrari et al. [27]. However the clinical translation of *ABCB1* genotyping for predicting atorvastatin-induced myopathy is unlikely, because although the findings by Hoenig et al. [41] and Ferrari et al. [27] are statistically significant, they offer little clinical discrimination.

### Pravastatin

Eight SIM clinical outcome studies included pravastatin. None of the SIM clinical outcome studies, however, included only pravastatin. In addition, the number of patients treated with pravastatin in the available studies was low. Therefore, attempts to teasing out the specific effects of pharmacokinetic genetic variants on pravastatin-induced myopathy are futile. The study by Donnelly et al. [32] found a significant association of *SLCO1B1* T521C with clinical outcome overall but did not perform pravastatin-specific analyses. The majority of patients in the Donnelly et al. [32] study were treated with simvastatin; therefore, the association may have been driven by simvastatin alone. Studies by Linde et al. [42], Brunham et al. [35], and Ruano et al. [43] included pravastatin, but they did not find a significant association for *SLCO1B1* variation overall or perform pravastatin-specific analyses. Voora et al. [23] published the only study that performed pravastatin-specific analyses, but they did not find a significant association with *SLCO1B1*. This negative clinical outcome data is surprising since pharmacokinetic studies have shown very large differences in pravastatin exposure by *SLCO1B1* T521C genotypes (232% difference in exposure between homozygotes) [44]. Because only one clinical outcome study performed a pravastatin-specific analysis, and the pharmacokinetic effect of *SLCO1B1* variation is large, *SLCO1B1* has the potential to be clinically important in pravastatin-induced myopathy. This is unlike atorvastatin, in which there is enough clinical outcome data currently available to rule out the clinical importance of *SLCO1B1* variation and atorvastatin-induced myopathy.

### Rosuvastatin

Nine SIM clinical outcome studies included rosuvastatin. *SLCO1B1* T521C is the only pharmacokinetic genetic variant reported to be associated with rosuvastatin-induced myopathy. This association was only found in the studies by Donnelly et al. [32] and Ferrari et al. [27], but the proportion and number of patients treated with rosuvastatin in these studies were very small (0.6%-3% of 4,196 patients in Donnelly et al. [32] and 22 in Ferrari et al. [27]). Simvastatin-treated patients were present in both studies; therefore the results could have been driven by the simvastatin-treated patients, but this cannot be determined because rosuvastatin-specific analyses were not performed. Danik et al. [45] performed the only study that focused specifically on rosuvastatin (n=8,872), and they did not confirm an association with *SLCO1B1* T521C. Notably, the patients studied by Danik et al. [45] were a highly selective patient population from the JUPITER trial [46], including only apparently healthy men aged 50 and older and women aged 60 and older with LDL-C levels of less than 130 mg/dL (3.4 mmol/L) and high-sensitivity C-reactive protein levels of 2.0 mg/L or higher. Eighty percent of patients that were screened for the JUPITER trial were ineligible. The rates of myopathy were similar in the rosuvastatin- and placebo-treated groups, bringing into question the generalizability of this finding to real-world clinical practice. The observational study by Puccetti et al. [33] included 30 patients treated with rosuvastatin, and in their rosuvastatin-specific analysis, *SLCO1B1* T521C was not associated. The studies by Linde et al. [42], Brunham et al. [35], and Ruano et al. [43] included patients on rosuvastatin, but they did not find a significant association for *SLCO1B1* variation overall and they did not perform rosuvastatin-specific analyses. Based on the currently available data, it seems that *SLCO1B1* variation is not important for predicting rosuvastatin-induced myopathy. This is in concordance with pharmacokinetic data. *SLCO1B1* T521C is associated with much larger increases in exposure for simvastatin (221% between homozygotes) than rosuvastatin (65% between homozygotes) [37].

### Other Statins

Of the 18 studies that we identified assessing pharmacokinetic genetic variants and SIM clinical outcome (Table 2), no evidence exists to support a specific association with the remaining statins: fluvastatin, lovastatin, or pitavastatin. The number of patients treated with fluvastatin or lovastatin in the SIM clinical outcomes studies was less than 10 in all studies, except for the study by Donnelly et al. [32] which had approximately 210 treated with fluvastatin. No studies performed specific analyses of these other statins, and none of the SIM clinical outcome studies included any patients treated with pitavastatin. Studies demonstrated an association between pharmacokinetic genetic variants and the pharmacokinetics of fluvastatin, lovastatin, and pitavastatin [47–49], but whether these differences in pharmacokinetics translate to differences in SIM clinical outcomes will need to be determined.

## Discussion

Statins are already the number one prescribed class of drugs in the US, and with the new guidelines on the treatment of cholesterol [4], the number of Americans treated with statins is expected to increase. Statins can be extremely effective, but many patients cannot tolerate statin therapy due to SIM. Therefore strategies to predict or mitigate SIM are critically needed. SIM is related to statin concentrations [12], and variants in pharmacokinetic genes affecting statin concentrations have been linked to SIM clinical outcome. The data to support *SLCO1B1* variation and simvastatin-induced myopathy was sufficiently strong to incite CPIC to write guidelines on the translation of *SLCO1B1* genotype into the clinical use of simvastatin [13]. These CPIC guidelines demonstrate an example of how pharmacokinetic genetic variants can be used in clinical practice. Specifically, these guidelines recommend that patients needing treatment with 40mg of simvastatin and carrying at least one copy of the decreased activity allele of *SLCO1B1* rs4149056 should be treated with a lower dose plus serial CK monitoring or an alternative statin. Our review evaluated the literature on other variants and statins, assessing their potential for clinical translation to predict SIM. Notably, other strategies for reducing SIM show promise, such as supplementation with coenzyme Q10 [50].

Eighteen studies of SIM clinical outcome and pharmacokinetic genetic variants were identified. For simvastatin, based on the currently available data, it seems unlikely that pharmacokinetic genes other than *SLCO1B1* will be clinically important for predicting risk of simvastatin-induced myopathy. Atorvastatin and rosuvastatin are also substrates for *SLCO1B1*, but the currently available data does not support the clinical translation of *SLCO1B1* for prediction of atorvastatin- or rosuvastatin-induced myopathy. It is currently unknown whether *SLCO1B1* could have utility for predicting lovastatin-induced myopathy because, to our knowledge, *SLCO1B1* variation has not been studied for an association with lovastatin-induced myopathy or pharmacokinetics. It is not likely that *SLCO1B1* genotype will be associated with fluvastatin-induced myopathy clinical outcome because *SLCO1B1* variation was not associated with variation in fluvastatin pharmacokinetics [44]. However, *SLCO1B1* may have potential to be clinically important in pravastatin and pitavastatin-induced myopathy. The pharmacokinetic differences for pravastatin and pitavastatin between *SLCO1B1* genotypes are very large and of the same magnitude as those for simvastatin [44,49]. Therefore, further studies of *SLCO1B1* and pravastatin and pitavastatin SIM clinical outcome are needed. The currently available literature does not support genes other than *SLCO1B1* and SIM clinical outcome, except for possibly *CYP2D6* and atorvastatin-induced myopathy. The association between *CYP2D6* and atorvastatin-induced myopathy was significant in both discovery and validation cohorts [21], but since it is unclear whether *CYP2D6* contributes to atorvastatin metabolism, mechanistic studies will be necessary before clinical translation could be considered.

Upon completion of our analysis and review of the current literature, we made three generalized observations that are noteworthy and may expedite investigators’ effort to translate SIM pharmacokinetic genetic research findings into clinical practice. The first major observation is that any study of pharmacokinetic genetic variants and SIM should perform statin-specific analyses. This is because the pharmacokinetic profiles for each individual statin are unique. For example, Zuccaro et al. [22] did not find a significant association between cytochrome P450 variants and SIM clinical outcome, but they included patients on pravastatin, which is not metabolized by cytochrome P450s. Even if statins are substrates for the same metabolizing enzymes or transporters, their specificities vary. For example, Ruano et al. [43] did not find a significant association of *SLCO1B1* variation with SIM clinical outcome, and the majority of patients were treated with simvastatin, atorvastatin, or rosuvastatin. All three drugs are transported by *SLCO1B1* but to varying degrees. The difference in exposure between *SLCO1B1* T521C homozygotes is 221% 144%, and only 65% for simvastatin, atorvastatin, and rosuvastatin, respectively [37]. The inclusion of statins other than simvastatin could be diluting the power to detect an association. Of note, this effect could happen in the opposite direction: the association with simvastatin could drive the association for other statins. For example, Donnelly et al. [32] found a significant association of *SLCO1B1* variation and SIM clinical outcome in patients on a variety of statins. The majority of patients were treated with simvastatin, but only simvastatin-specific analyses were performed. Therefore, it cannot be determined whether the association is present for other statins.

The second major observation is the lack of multi-variant analyses. Multi-variant analyses are necessary because multiple genes are involved in the pharmacokinetics of statins. For example, simvastatin is not only a substrate for *SLCO1B1*, but also *CYP3A4* and *CYP3A5*, which also have genetic variants that can affect their function. Because functional variants in these genes are common, it is possible that a given patient possesses decreased function alleles for all three of those genes. The clinical implications of that situation are currently unknown, and the additive effects of multiple variants and gene-gene interactions need to be assessed. Many of the published studies tested multiple pharmacokinetic genetic variants, but the variants were assessed only on an individual basis. Mechanistic data supports the potential for gene-gene interactions. The expression of *CYP3A4* and *CYP3A5*, for example, may be co-regulated [51]. Further research will also be necessary to assess the incorporation of multiple non-pharmacokinetic genes (such as the gene for the LDL receptor) into multi-variant analyses. The power of traditional statistical methods to detect genetic associations and gene-gene interactions declines rapidly as the number of variants tested is increased. Therefore, novel analytical approaches (*e.g*., machine learning) may be necessary for large multivariant analyses.

Our final observation on this body of research is the definition of SIM widely varied across studies. The spectrum of SIM clinical outcome definitions ranged from a purely biochemical definition (*e.g.,* Brunham et al. [35] defined SIM as plasma CK values > 10× the upper limit of normal without regard to symptoms) to a purely symptomatic definition (*e.g.,* Hoenig et al. [41] defined SIM as muscle pain, tenderness, or weakness during the treatment period). Some studies used a composite endpoint that included both biochemical and symptomatic definitions (*e.g.,* Voora et al. [23] defined a composite adverse event including discontinuation for any side effect, myalgia/muscle cramps, or CK >3× the upper limit of normal during follow-up). These varying definitions of SIM may have contributed to inconsistent results across SIM clinical outcome studies and make comparisons across studies extremely difficult. Because symptoms of SIM do not correlate well with biochemical definitions, developing a standard definition of SIM for future research may be the most difficult task to achieve going forward. However, *any* adverse effect contributing to patient intolerance or prescribing changes should be considered clinically relevant.

## Conclusion

In conclusion, no other pharmacokinetic genes (besides *SLCO1B1* for simvastatin-induced myopathy) are currently ready for clinical translation. With additional data, however, *SLCO1B1* variation may have potential to be clinically relevant for pravastatin- and pitavastatin-induced myopathy and *CYP2D6* variation may be clinically relevant for atorvastatin-induced myopathy. The following should be considered in future research efforts aiming to advance our understanding of variants in pharmacokinetic genes and SIM: statin-specific analyses, multi-variant analyses, and standardizing a definition for SIM clinical outcome. Notably, this review is a single snapshot in a continuum of ongoing research, and the translation of pharmacokinetic genetic variants into clinical practice is an immensely complicated task that will require significant additional investments of resources to achieve. This research endeavor should remain a high priority as cardiovascular disease, statin use, and SIM are pervasive in our health care system. If pharmacokinetic genetic variants could be used to aid clinical decision-making and improve SIM clinical outcomes, the impact on public health could be substantial.

## Figures and Tables

**Table 1:** Candidate genes involved in statin pharmacokinetics considered for this review.

Gene abbreviation	Statin metabolizing enzyme or transporter
*ABCB1*	ATP-binding cassette, sub-family B, member 1
*ABCB11*	ATP-binding cassette, sub-family B, member 11
*ABCC2*	ATP-binding cassette, sub-family C, member 2
*ABCG2*	ATP-binding cassette, sub-family G, member 2
*CYP2C8*	cytochrome P450, family 2, subfamily C, polypeptide 8
*CYP2C9*	cytochrome P450, family 2, subfamily C, polypeptide 9
*CYP2C19*	cytochrome P450, family 2, subfamily C, polypeptide 19
*CYP2D6*	cytochrome P450, family 2, subfamily D, polypeptide 6
*CYP3A4*	cytochrome P450, family 3, subfamily A, polypeptide 4
*CYP3A5*	cytochrome P450, family 3, subfamily A, polypeptide 5
*SLC15A1*	solute carrier family 15 (oligopeptide transporter), member 1
*SLC22A6*	solute carrier family 22 (organic anion transporter), member 6
*SLC22A8*	solute carrier family 22 (organic anion transporter), member 8
*SLCO1A2*	solute carrier organic anion transporter family, member 1A2
*SLCO1B1*	solute carrier organic anion transporter family, member 1B1
*SLCO1B3*	solute carrier organic anion transporter family, member 1B3
*SLCO2B1*	solute carrier organic anion transporter family, member 2B1
*UGT1A1*	UDP glucuronosyltransferase 1 family, polypeptide A1
*UGT1A3*	UDP glucuronosyltransferase 1 family, polypeptide A3
*UGT2B7*	UDP glucuronosyltransferase 2 family, polypeptide B7

ATP=Adenosine triphosphate; UDP=Uridine diphosphate

**Table 2:** List of reviewed studies meeting inclusion & exclusion criteria by publication year.

Reference	N	Study design	Statins	Statin PK genes
[20]	88	Prospective trial	simvastatin	*CYP2D6*
[38]	137	Case-control	atorvastatin	*CYP3A4* *CYP3A5*
[26]	146	Prospective trial	simvastatin	*ABCB1* *CYP3A4* *CYP3A5*
[12]	28	Case-control	atorvastatin	*ABCB1* *SLCO1B1* *CYP3A5*
[22]	100	Case-control	simvastatin fluvastatin pravastatin atorvastatin rosuvastatin	*CYP3A5* *CYP2C9* *CYP2D6*
[21]	263	Case-control and longitudinal study	atorvastatin simvastatin	*CYP2D6* *CYP3A4* *CYP3A5* *CYP2C9* *CYP2C8*
[18]	16,839	Case-control	simvastatin	GWAS
[23]	452	Prospective, randomized trial	atorvastatin simvastatin pravastatin	*CYP2D6* *CYP2C8* *CYP2C9* *CYP3A4* *SLCO1B1*
[33]	76	Case-control	atorvastatin rosuvastatin	*SLCO1B1*
[42]	46	Retrospective cohort	atorvastatin pravastatin simvastatin lovastatin rosuvastatin fluvastatin	*SLCO1B1*
[41]	98	Retrospective cohort	atorvastatin	*ABCB1*
[43]	793	Cross-sectional	simvastatin atorvastatin rosuvastatin pravastatin lovastatin fluvastatin	*SLCO1B1*
[30]	399	Case-control	atorvastatin simvastatin cerivastatin pravastatin	GWAS
[32]	4,196	Observational cohort	simvastatin atorvastatin pravastatin fluvastatin cerivastatin rosuvastatin	*SLCO1B1*
[35]	109	Case-control	simvastatin atorvastatin pravastatin rosuvastatin	*SLCO1B1*
[45]	8,782	Sub-study of clinical trial	rosuvastatin	*SLCO1B1*
[34]	488	Case-control	simvastatin atorvastatin cerivastatin pravastatin rosuvastatin fluvastatin	*SLCO1B1*
[27]	66	Case-control	atorvastatin rosuvastatin simvastatin	*ABCB1* *ABCG2* *SLCO1B1*
